# Plasma Lipoprotein-associated Phospholipase A2 and Superoxide Dismutase are Independent Predicators of Cognitive Impairment in Cerebral Small Vessel Disease Patients: Diagnosis and Assessment

**DOI:** 10.14336/AD.2019.0304

**Published:** 2019-08-01

**Authors:** Shuzhen Zhu, Xiaobo Wei, Xiaohua Yang, Zifeng Huang, Zihan Chang, Fen Xie, Qin Yang, Changhai Ding, Wei Xiang, Hongjun Yang, Ying Xia, Zhong-Ping Feng, Hong-Shuo Sun, Midori A Yenari, Lin Shi, Vincent CT Mok, Qing Wang

**Affiliations:** ^1^Department of Neurology and; ^2^Department of Orthopedics, Zhujiang Hospital of Southern Medical University, Guangdong, China.; ^3^Menzies Institute for Medical Research, University of Tasmania, Hobart, Australia.; ^4^Department of Neurology, Guangzhou General Hospital of Guangzhou Military Command, Guangdong, China.; ^5^Shanghai Key Laboratory of Acupuncture Mechanism and Acupoint Function, Fudan University, Shanghai, China.; ^6^Department of Physiology and; ^7^Department of Surgery, Faculty of Medicine, University of Toronto, Toronto, Ontario M5S 1A8, Canada.; ^8^Department of Neurology, University of California, San Francisco & the San Francisco Veterans Affairs Medical Center, San Francisco, USA.; ^9^Department of Imaging and Interventional Radiology, The Chinese University of Hong Kong, Hong Kong, China.; ^10^BrainNow Research Institute, Shenzhen, China.; ^11^Gerald Choa Neuroscience Centre, Department of Medicine and Therapeutics, Faculty of Medicine, Prince of Wales Hospital, The Chinese University of Hong Kong, Hong Kong, China.

**Keywords:** Lp-PLA2, superoxide dismutase, cerebral small vessel disease, cognition

## Abstract

Lipoprotein-associated phospholipase A2 (Lp-PLA2) and superoxide dismutase (SOD) are linked to regulating vascular/neuro-inflammation and stroke. Using a retrospective design, we investigated whether circulating Lp-PLA2 and SOD in cerebral small vessel disease (CSVD) patients were associated with cognitive impairment. Eighty-seven CSVD patients were recruited. Plasma Lp-PLA2 and SOD were determined, and cognitive status was measured by the Mini-Mental State Examination (MMSE) and Montreal Cognitive Assessment (MoCA). The severity of white matter hypoerintensities (WMHs) in CSVD patients was rated according to Fazekas scales, and Lp-PLA2/SOD levels and MMSE/MoCA were compared. Multiple linear regressions were used to evaluate the relationship between Lp-PLA2 and SOD and the cognitive impairment. Ordinal logistic regression and generalized linear models (OLRGLMs) were applied to confirm whether Lp-PLA2 and SOD are independent risk factors for cognitive impairment in CVSD. Lp-PLA2 and SOD with mild or severe cognitive impairment were lower than those with normal congnition. Lp-PLA2 and SOD in CSVD patients with severe WMHs were significantly lower than those with mild or moderate WMH lesions. We noted positive linear associations of Lp-PLA and SOD with cognitive impairment in CSVD, independent of LDL-C. OLRGLMs confirmed that Lp-PLA2 and SOD were independent risk factors of cognitive impairment in CSVD. Lp-PLA2 and SOD are independently associated with cognitive impairment and WMH lesion, and may be useful for the rapid evaluation of cognitive impairment in CSVD. Lp-PLA2/SOD are modifiable factors that may be considered as therapeutic targets for preventing cognitive impairment in CSVD.

Cerebral small vessel disease (CSVD) is a highly prevalent condition associated with neuroinflammation and cognitive impairment [1, 2]. However, the diagnosis and classification of CSVD with cognitive impairment were somewhat difficult for community doctors or non-Stroke subspecialist. Clinically, there is no blood marker available to diagnose and assess CSVD with cognitive impairment in clinical settings [3, 4]. Worldwide, the number of CSVD patients is more common in Asians than in Caucasians [5]. Growing evidence has shown that systemic inflammatory mediators such as superoxide dismutase (SOD) and lipoprotein-associated phospho-lipase A2 (Lp-PLA2) are associated with CSVD and cognitive impairment [4, 6-8]. However, the role of Lp-PLA2 and SOD, especially SOD, in CSVD patients with cognitive impairment is largely unknown.

Lp-PLA2 acts as an enzyme and proinflammatory marker of cardio- and cerebrovascular risk; however, some studies have indicated that Lp-PLA2 exerts antioxidative and anti-inflammatory functions under certain circumstances [9]. Lp-PLA2 circulates in plasma in its active form as a complex with low-density lipoprotein (LDL) and high-density lipoprotein (HDL). SOD is an enzyme that catalyzes the dismutation of the superoxide radical into H_2_O_2_ and oxygen and has powerful anti-inflammatory activity [10, 11]. A recent study in humans suggested that plasma SOD could be a marker of vascular alterations. Other studies have also indicated that higher levels of Lp-PLA2 are associated with cerebral ischemic stroke and cognitive impairment [7, 8, 11]; however, the roles of SOD in cognitive impairment remain unexplored.

To explore the association of Lp-PLA2 and SOD with CSVD patients with cognitive impairment, we asked whether (1) plasma Lp-PLA2 and SOD levels in CSVD patients with cognitive impairment were higher or lower than those in CSVD patients with normal cognition [NC], (2) there was a certain linear relationship between Lp-PLA2/SOD and cognitive impairment in CSVD, and (3) the Lp-PLA2/SOD-related receiver operating characteristic (ROC) curve could be used to distinguish CSVD with mild or severe cognitive impairment [MCI, SCI] from those with NC.

MATERIALS AND METHODS

## Study design

This study was a retrospective analysis of collected data from patients admitted with subcortical white matter hyperintensity (WMH) and lacunar infarct in two medical centers (Zhujiang hospital and Guangzhou General Hospital of Military Command, Guangzhou, China). The study was approved by the ethics Human Research Committee at Zhujiang Hospital of Southern Medical University and complied with the principles outlined in the revised Declaration of Helsinki of 1975 and the National Institutes of Health Human Subjects Policies and Guidelines released in 1999. Informed written or verbal consent was obtained from patients and family members.

**Figure 1.**
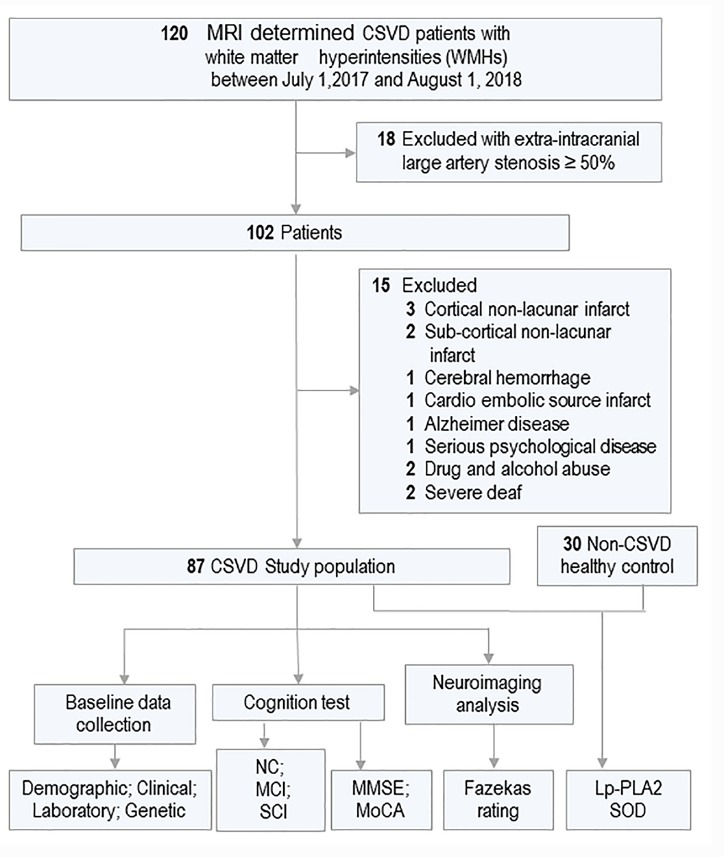
All 120 CSVD patients who met the clinical diagnostic criteria of white matter hyperintensities were enrolled in this study from Jul 2017 to Aug 2018 Of 33 participants met the excluded criteria and 87 participants were included in this study for further analyzing. 30 age and gender-matched non-CSVD healthy controls (HC) were enrolled from physical examnination center. The plasma level of Lp-PLA2 and SOD was compared between CSVD and HC groups. In addition, The cognitive function and white matter hyperintensity were evaluated and the variables were collected and analyzed among groups.

### Patient selection

One-hundred and twenty consecutive CSVD participants with Magnetic Resonance Imaging (MRI)-determined subtypes (WMH and lacunar infarcts) were recruited from July 2017 to August 2018. All participants were clinically diagnosed with CSVD at Zhujiang hospital and Guangzhou General Hospital of Military Command based on the criteria of Trial of Org 10172 in Acute Stroke Treatment [12]. All participants underwent T2, T1 turbo field echo, fluid-attenuated inversion recovery (FLAIR) imaging using a 3.0-T MRI scanner (Philips 3.0T Achieva; Philips Healthcare, Andover, MA). Subcortical WMHs were defined according to the study of Mok [13]. A lacune was defined as a round or ovoid lesion ≥3 mm and ≤15 mm in diameter found on T1-weighted and T2-weighted images with a perilesional halo on FLAIR images as defined by Wardlaw et al [14]. The study flow diagram was shown in Figure 1. Of the 120 participants, we excluded 18 patients who showed extra-intracranial large artery stenosis ≥50% and 3 who exhibited a cortical nonlacunar infarct. Others with subcortical nonlacunar infarct (n=2), cerebral hemorrhage (n=1), cardio embolic source infarct (n=1), Alzheimer disease (AD; n=2), serious psychological disease (n=2), drug and alcohol abuse (n=2), severe deaf (n=2), were also excluded from this study. Thus, the final analysis was performed in 87 CSVD eligible participants. In addition, 30 age-and gender-matched healthy control (HC) subjects were recruited at the physical examination center and the plasma levels of Lp-PLA2 and SOD were collected. We received written informed patient consent to perform this study.

### Participant characteristics

Eligible participant characteristics that were regarded as likely relevant confounders of cognition or Lp-PLA2 were recorded as baseline data. In this study, the following variables were collected: 1) demographic data (gender, age, years of education); 2) clinical data (body mass index [BMI], history of hypertension, diabetes and coronary heart disease [CHD], medical history of statin and antithrombotic use), with hypertension and diabetes mellitus defined as a self-reported medical diagnosis, antihypertensive/antidiabetic medication use, or a new diagnosis according to increased fasting and postprandial blood glucose, or CHD defined as a self-reported medical diagnosis or heart disease medication use, and BMI calculated as weight divided by height squared (kg/m^2^); 3) laboratory markers such as cholesterol metabolism-relevant biomarkers including LDL-cholesterol (LDL-C), HDL-cholesterol (HDL-C) and total cholesterol, kidney function-relevant biomarkers including urea, creatinine (Cr) and UA, and ROS markers including Lp-PLA2 and SOD; 4) genetic data such as apolipoprotein E (APOE) allele carrier status.

### Cognitive impairment severity grading

The diagnosis of cognitive impairment was performed by two neruologists according to the criteria proposed by Philip B., which was recommeded by Guidelines for Cerebral Vascular Diseases[15]. Cognitive domains including executive/attention, memory, language, and visuospatial functions were assessed. A decline in cognitive function from a prior baseline in at least one cognitive domain and normal or mildly impaired instrumental activities of daily living were defined as MCI. A deficit in performance in ≥2 cognitive domains that are of sufficient severity to affect the subject’s activities of daily living was defined as SCI. The Mini-Mental State Examination (MMSE) and Montreal Cognitive Assessment (MoCA) were evaluated on the day of administration.

### Laboratory biomarker measurements

Total cholesterol, HDL-C, LDL-C, urea, leukocyte, neutrophile granulocyte (NEU), hypersensitive C-reactive protein (hs-CRP), erythrocyte sedimentation rate (ESR), Cr, UA, Lp-PLA2, APOE and SOD in the plasma were measured twice during hospitalization. Fasting blood samples were collected in tubes containing EDTA and centrifuged at 3000 g for 10 min, and aliquots of plasma were stored at -70°C until use for biochemical analyses. Since the Lp-PLA2 mass test only detects a portion of the total Lp-PLA2, mainly the Lp-PLA2 associated with HDL, which is the “good cholesterol” that displays protective effects [16], and we wanted to explore the neuroprotective effects of plasma Lp-PLA2, therefore here we only measured Lp-PLA2 mass in the current study. Lp-PLA2 mass was detected with the Human PLA2G7/PAF-AH/Lp-PLA2 Quantikine Enzyme-linked Immunosorbent Assay (ELISA) Kit (Bio-Techne Corporation, R&D system, Minnesota) according to the referenced instructions by Packard et al. and the manufacturer’s instructions [17]. Briefly, 10 µL EDTA plasma was added to a 96-well plate precoated with an antibody specific to Lp-PLA2. After incubation with the Lp-PLA2-specific primary and biotin-conjugated anti-bodies, the plasma concentration of Lp-PLA2 was determined by comparing the optical density (O.D.) of the samples to the standard curve. Plasma SOD levels were measured with a Cu/Zn SOD ELISA kit (Bio-Techne Corporation, R&D system, Minnesota, USA) according to the manufacturer’s instructions. Absorbance was read on a spectrophotometer (Thermo Luminoskan Ascent, Waltham MA, USA) at 450?nm [10].

APOE genotyping was detected by Q-PCR as described by Christensen et al., [18]. Genomic DNA was extracted from blood samples using Qiagen DNA Blood kits (#51162; Qiagen Inc., Valencia, CA, USA). SNP-specific primers and probes were designed by Invitrogen (Invitrogen,). TaqMan real-time PCR assays were performed in an ABI 7900 HT machine, using the following cycling program: 95°C for 10 min followed by 40 cycles of 95°C for 15 sec and 60°C for 1 min.

**Figure 2.**
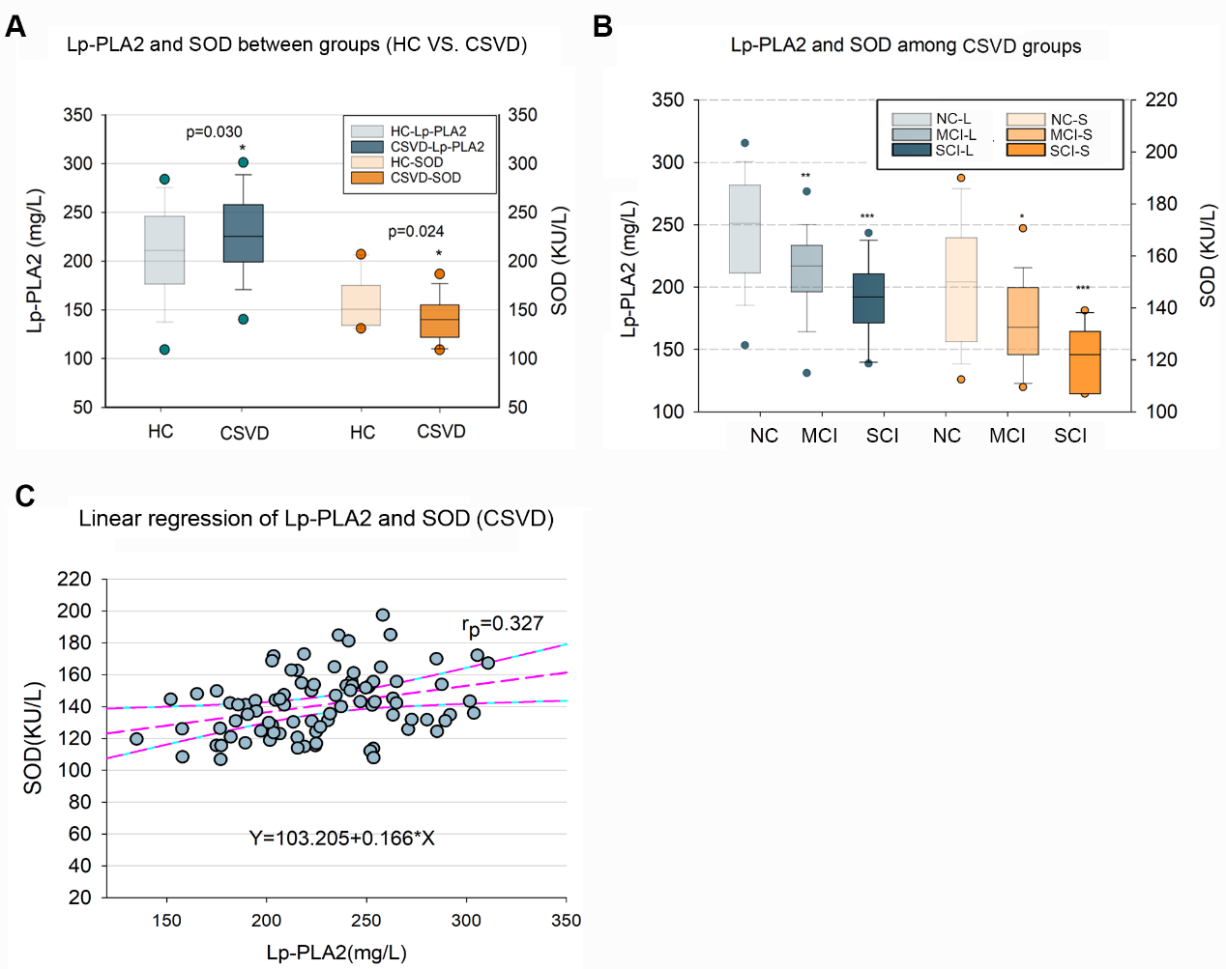
Comparison of biomarkers for Lp-PLA2 and SOD levels in the CSVD patients with different severity of cognitive impairment (**A**) Comparison was made using independent t-test between the CSVD patients and HC (healthy control). The level of Lp-PLA2 increased and SOD decreased in CSVD patients. (**B**) Comparisons were made using one-way covariance with adjusted for the confounders (sex, age, education, BMI, cholesterol, HDL-C, LDL-C, urea, Cr, UA, hypertension, diabetes, CHD, APOE genotype, and medication use) followed by a post hoc Bonferroni test. Cognitive impairment group (MCI and SCI) displayed significantly lower levels of Lp-PLA2 and SOD than the normal cognition (NC) group (see also table 1). (**C**) The plasma Lp-PLA2 was linear correlated with SOD (r=0.327, p <0.05). Abbreviations: APOE, apolipoprotein E; BMI, body mass index; CHD, coronary heart disease; Cr, creatinine; CSVD, cerebral small vessel disease; HC, healthy control; HDL-C, High-density lipoprotein cholesterol; LDL-C, low-density lipoprotein cholesterol; Lp-PLA2, Lipoprotein-associated Phospholipase A2; MCI: mild cognitive impairment; MMSE, Mini-Mental State Examination; MoCA, Montreal Cognitive Assessment; NC, normal cognition; SCI, severe cognitive impairment; SOD, Superoxide Dismutase; UA, uric acid. * Compared to NC *p* <0.05, ** Compared to NC *p* <0.01, *** Compared to NC *p* <0.001, ^##^ Compared to MCI *p* <0.01.

### The rating of white matter hyperintensities with Fazekas scales

MRI including T2 and FLAIR sequences were used in this study. The severity of white matter hypoerintensities (WMHs) was rated according to Fazekas scales by two raters. [19] According to the scores of Fazekas, the CSVD patients were divided into three groups (1: Mild WMHs, 2: Modest WMHs, 3, Severe WMHs).

### Statistical Analysis

All data analyses were conducted using SPSS 20.0 (IBM Corporation, Armonk, NY, USA). A ^*^p-value <0.05 was considered significant. All continuous variables, for example age, BMI, MoCA score, MMSE score, and Lp-PLA2, SOD, UA and lipid concentrations, are shown as the mean ± SD; all categorical variables, such as gender, APOE genotype, hypertension, and diabetes, are presented as a percentage. Significant differences in Lp-PLA2 and SOD between healthy and CSVD subjects were analyzed with independent *t*-test; while differences in plasma Lp-PLA2 and SOD levels among CSVD patients with different degrees of cognitive impairment, and differences in Lp-PLA2 and SOD levels among CSVD patients with different degrees of WMHs were analyzed with one-way covariance followed by a post hoc Bonferroni test. Pearson correlation analysis was used to examine the relationship between Lp-PLA2 and other risk factors. Multiple linear regressions with a backward elimination model were used to detect the adjusted partial linear correlation between Lp-PLA2/SOD and MMSE/MoCA scores. Ordinal logistic regression and generalized linear models (OLRGLMs) were used to detect the significantly independent associated factors for cognitive decline in CSVD. To assess the diagnostic value of Lp-PLA2 and SOD in the identification of CSVD with MCI or SCI and discrimination from CSVD with NC, ROC analysis was conducted, and the ROC curve for the combination of Lp-PLA2 and SOD was calculated to screen for a better diagnosis tool.

**Table 1:** Baseline Characteristic of Study CSVD Population.

Variable	Subgroup comparison				
	NC (N= 46)	MCI (N=30)	SCI (N=11)	F or X^2^ *	p value
Demographic					
Age, mean (SD), y	63.9(10.1)	67.9 (12.1)	74.2(13.7)	4.039	.021#
Male, N (%)	29 (63.4)	19 (63.3)	8 (72.7)	0.384	.902
Education, mean (SD), y	9.0 (3.4)	7.5 (2.1)	6.6 (2.7)	4.243	.018##
Clinical					
BMI, mean (SD)	23.9(2.8)	23.1(3.2)	24.0(4.5)	0.600	.551
Hypertension, N (%)	28 (60.9)	16 (53.3)	9 (81.80)	2.743	.274
Diabetes, N (%)	13(28.3)	7(23.3)	4(36.4)	0.706	.706
CHD, N (%)	20 (43.5)	11(36.7)	7 (63.6)	2.381	.301
Statin Use, N (%)	43 (93.5)	24 (80.0)	9 (81.8)	3.336	.165
Antithrombotic use, N (%)	44 (95.7)	24 (80.0)	9 (81.8)	4.926	0.072
Laboratory mean (SD)					
Cholesterol, (mmol/L)	4.8±1.0	4.4±1.0	4.1±1.1	2.874	.062
HDL-C, (mmol/L)	1.3±0.5	1.2±0.4	1.2±0.2	0.621	.540
LDL-C, (mmol/L)	2.7±0.9	2.6±0.7	2.5±0.9	0.561	.573
Urea, (mmol/L)	5.3±1.4	6.0±1.5	6.0±2.9	1.909	.155
Cr, (umol/L)	78.6±28.1	84.2±34.1	97.7±36.5	1.690	.191
UA, (umol/L)	408.8±115.0	384.3±110.2	386.3±122.2	0.478	.622
Leukocyte, (G/L)	6.8±1.8	7.6±2.4	7.0±1.5	1.456	0.239
NEU, (G/L)	4.2±1.4	4.9±2.2	4.8±1.4	1.702	0.189
NEU	60.8±9.0	62.7±9.9	67.3±7.3	2.256	0.111
Hs-CRP, (mg/L)	3.4±4.1	4.4±10.2	3.0±2.0	0.143	0.867
ESR, (mm/H)	22.4±17.0	30.2±28.7	23.6±18.3	0.752	0.476
Lp-PLA2 mass, (mg/L)	246.2 ±45.4	212.4±35.3	189.2±30.7	11.82	<0.001***
SOD, (KU/L)	149.6±24.3	135.4±16.4	120.7± 12.3	10.354	<0.001***
Genetic					
APOE4 carrier, No. (%)	6 (13.0)	3(10.0)	1(9.1)	0.237	.897

**Figure 3.**
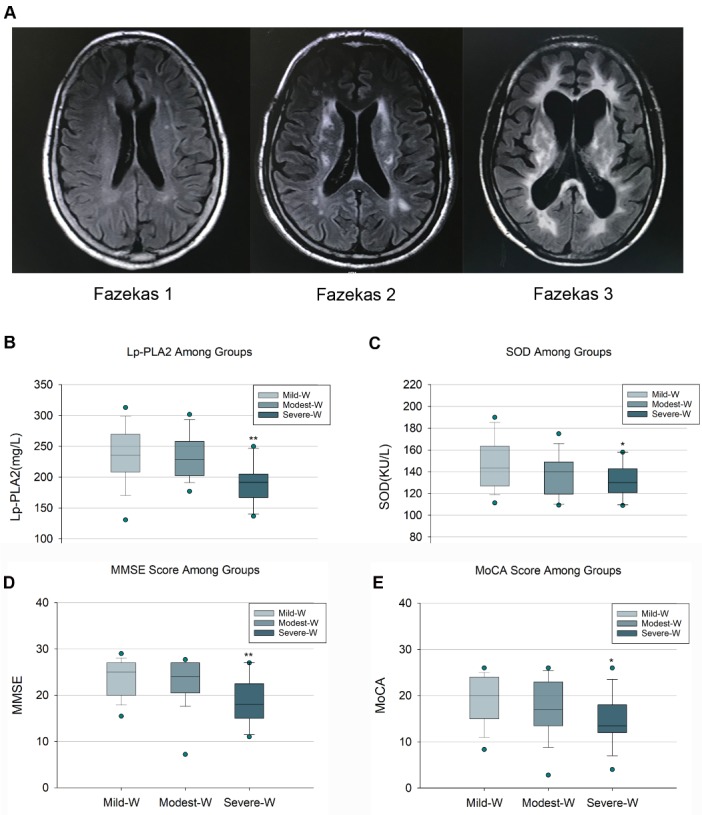
Comparison of Lp-PLA2 and SOD in the CSVD patients with different severity of WMHs (**A**) The representative MRI imagings of WMHs in CSVD patients with different severity classified by Fazekas Scores. The levels of Lp-PLA2 (B) and SOD (C) were relatively lower in the CSVD patients of Severe-W compared to those in the Mild-W. The scores of MMSE (D) and MoCA (E) were lower in patients in the Severe-W group compared to those in the Mild-W group. Abbreviations: CSVD, cerebral small vessel diseases; MMSE, Mini-Mental State Examination; Mild-W, Mild-White matter hyperintensities, MoCA, Montreal Cognitive Assessment; Modest-W, Modest- White matter hyperintensities, MRI, Magnetic Resonance Imaging; Severe-W, Severe-White matter hyperintensities, SOD, Superoxide Dismutase; WMHs, White matter hyperintensities. * Compared to Mild-W *p* <0.05, ** Compared to Mild-W *p* <0.01.

Data shown are mean ± S.D. The comparisons of Lp-PLA2 and SOD among the groups were done with one-way covariance with adjusted for the confounders (see also Fig. 3). Comparisons of confounders were made using one-way ANOVA followed by Bonferroni post-hoc tests. Values with *p*<0.05 was regarded as significant related to multiple comparisons. Abbreviations: NC, normal cognition; MCI: mild cognitive impairment; SCI, severe cognitive impairment; MMSE, Mini-Mental State Examination (range, 0-30, with 30 a perfect score); BMI, body mass index; CHD, coronary heart disease; HDL-C, High-density lipoprotein cholesterol; LDL-C, low-density lipoprotein cholesterol; Cr, creatinine ; UA, uric acid; Che, Cholinesterase; APOE, apolipoprotein E. Lp-PLA2, Lipoprotein-associated Phospholipase A2; SOD, Superoxide Dismutase; NEU, Neutrophile granulocyte; hs-CRP, hypersensitive C-reactive protein; ESR, Erythrocyte sedimentation rate.

# Compared to NC *p* = 0.023,

## Compared to NC *p* = 0.05

## RESULTS

### Participant characteristics

Eighty-seven participants were successfully screened and enrolled in the study, and neuropsychological test (MMSE and MoCA) evaluations were acquired for each participant. Forty-six of the patients with CSVD had NC (MMSE score exceeded 24 points), whereas 30 of the CSVD patients were classified as having MCI (MMSE score ranged from 18 to 23) and 11 participants as having SCI with an MMSE score no more than 17 points. Patients characteristics, including sex, age, BMI, past medical history (hypertension, diabetes and heart disease), plasma biomarkers, medication history (antithrombotic, lipid-regulating drugs), biomarkers of systemic inflammation (leukocyte, hs-CRP, neutrophile granulocyte, ESR) and APOE genotype, were summarized and included in this study (Table 1). Interestingly, besides significant differences were found among the groups in age (F=4.039, *p*=0.021) and years of education (F=4.243, *p*=0.018), we noted signifcant differences of Lp-LPA2 (F=11.82, *p*<0.001) and SOD (F=10.354, *p*<0.001) among NC, MCI and SCI groups. However, no significant differences in BMI, cholesterol, HDL-C, LDL-C, leukocyte, hs-CRP, neutrophile granulocyte, ESR, Urea, Cr, UA, sex, hypertension, diabetes, CHD, APOE genotype, or medication use among three groups were observed in this study.

### Comparison of plasma Lp-PLA2 and SOD levels in CSVD patients

Plasma Lp-PLA2 and SOD levels in CSVD patients across all groups were higher than those in HC (Fig. 2A). Analysis of covariance showed significant differences in Lp-PLA2 and SOD levels among the CSVD groups (SCI, MCI and NC) after adjusting other confounders for sex, age, education, BMI, cholesterol, HDL-C, LDL-C, urea, Cr, UA, hypertension, diabetes, CHD, APOE genotype, and medication use (F=11.82, *p*<0.001 for Lp-LPA2; F=10.354, *p*<0.001 for SOD, Table 1). Bonferroni post hoc comparisons showed that there were significant differences in Lp-PLA2 and SOD levels between the NC and MCI (*p*=0.002 for Lp-PLA2; *p*=0.013 for SOD) and between the NC and SCI groups (*p*<0.001 for Lp-PLA2; *p p*<0.001 for SOD, Fig. 2B). To evaluate whether there was a relationship between Lp-PLA2 and SOD levels, Pearson’s correlation between plasma Lp-PLA2 and SOD levels was analyzed. Lp-PLA2 levels were positively correlated with SOD levels (r=0.327, *p*<0.001), showing a linear relationship (Fig. 2C).

**Table 2:** Ordinal logistic regression of possible variables associated with cognitive decline in CSVD.

Variable	OR	
	Adjusted OR (95% CI)	p value
Age (years)	1.050 (0.991-1.120)	0.097
BMI	0.887 (0.715-1.100)	0.275
Education (years)	0.693 (0.528-0.908)	0.008**
Hypertension	0.652(0.154-2.759)	0.561
Diabetes, n (%)	2.176(0.450-10.525)	0.334
CHD, n (%)	2.236(0.522-9.528)	0.278
Cholesterol (mmol/L)	0.562(0.265-1.195)	0.134
HDL cholesterol (mmol/L)	0.966 (0.182-5.109)	0.080
LDL cholesterol (mmol/L)	2.601(0.942-7.184)	0.065
Urea (mmol/L)	1.521(1.015-2.280)	0.042
Cr (umol/L)	1.002(0.981-1.024)	0.848
UA (umol/L)	1.002(0.996-1.008)	0.466
SOD (KU/L)	0.933 (0.895-0.972)	0.001**
Lp-PLA2 mass (mg/L)	0.977 (0.962-0.992)	0.003**
Antithrombotic use, n (%)	8.069(0.327-199.032)	0.202
Statin use, n (%)	0.871(0.048-15.196)	0.925

Lower levels of education (*p*=0.008), relatively lower levels of SOD (*p*=0.001) and Lp-PLA2 (*p*=0.003) were independent associated factors of cognitive decline in patients with CSVD.

### Comparison of plasma levels of Lp-PLA2 and SOD in CSVD with different severity of WMHs

Both MMSE (F=5.582, *p*<0.01) and MoCA (F=3.488, *p*<0.05) scores significantly decreased in severe WMHs patients as showed in Figure 3D and E. According to the scores of Fazekas, the CSVD patients were divided into three groups as classified by the severity of WMH (1: Mild WMHs, 2: Modest WMHs, 3, Severe WMHs), which was shown in Figure 3A. The relatively lower levels of Lp-PLA2 (F=6.522, *p*<0.01) and SOD (F=3.396, *p*<0.05) were observed in severe WMHs subjects compared to those in the mild group (Fig. 3B, C).

### Linear correlation between plasma Lp-PLA2/SOD levels and MMSE or MoCA scores

**Figure 4.**
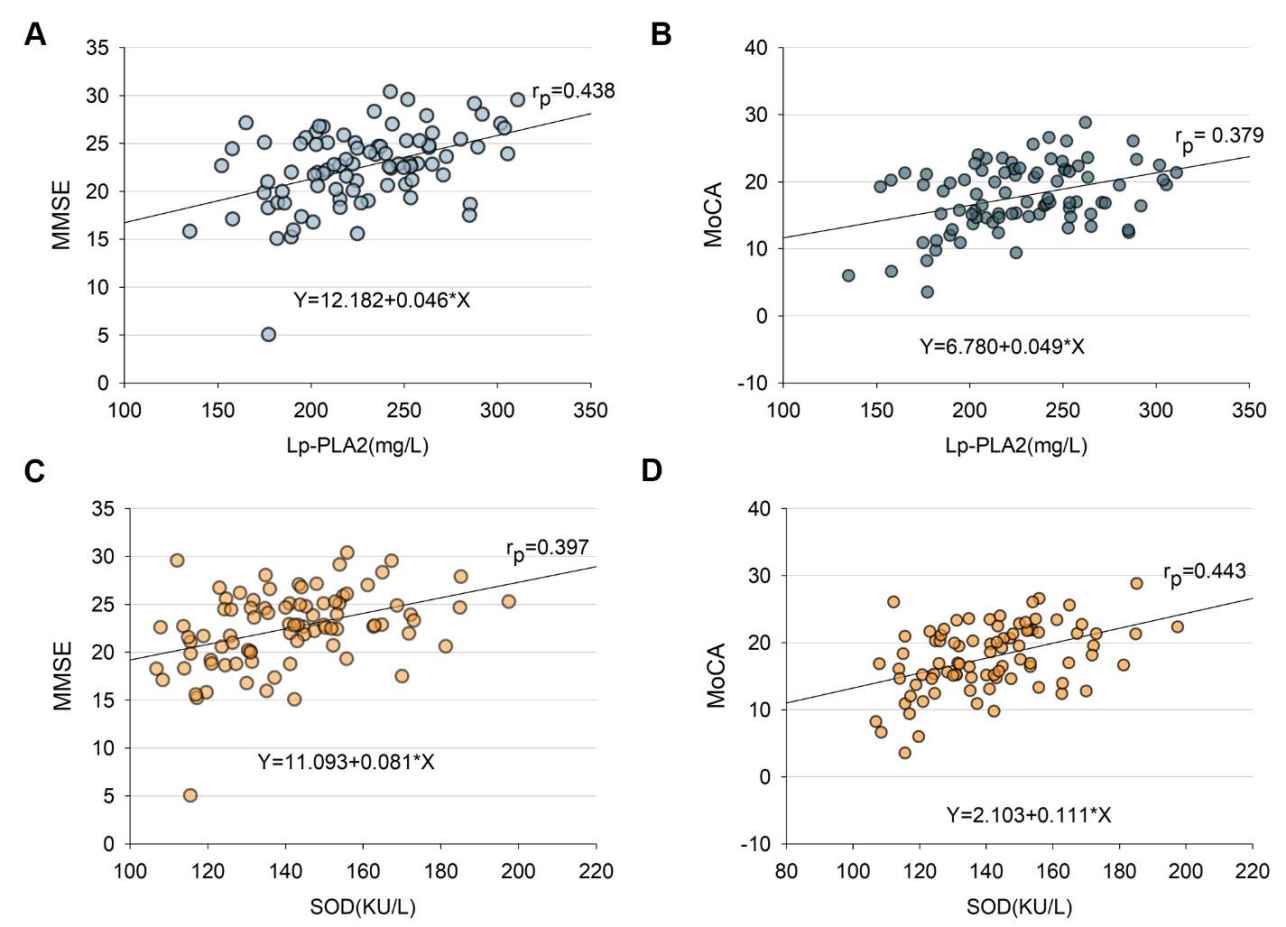
Linear correlation of plasma Lp-PLA2 and SOD with MMSE or MoCA (**A**) Lp-PLA2 level in plasma samples were measured. After adjusting for confounders, Lp-PLA2 linear correlated with cognitive impairment severity as measured by MMSE scores (r=0.438, *p* <0.001). (**B**) A similar trend was observed between SOD and MMSE score, and the correlation was statistically significant (r=0.379, *p* <0.01). (**C**) After adjusting for confounders, Lp-PLA2 linear correlated with cognitive impairment severity as measured by MoCA scores (r=0.397, *p* <0.001). (**D**) A similar trend was observed between SOD and MoCA score, and the correlation was statistically signficant (r=0.443, *p*<0.001). Abbreviations: Lp-PLA2, Lipoprotein-associated Phospholipase A2; MMSE, Mini-Mental State Examination; MoCA, Montreal Cognitive Assessment; SOD, Superoxide Dismutase.

The results from the backward elimination for multiple linear regression with adjustment for LDL-C and other parameters including age, gender, degree of education, hypertension, diabetes mellitus, CHD, statin use, antithrombotic use, HDL-C, cholesterol, urea, and Cr are presented in Supplementary Table 1 and Figure 4. The correlations between Lp-PLA2 and MMSE, and between SOD and MMSE were analyzed (Pearson correlation coefficient: 0.438 and 0.397, respectively, Fig. 4A, C). Linear regression analysis (Fig. 4A, C) showed that there was a linear relationship between Lp-PLA2 and MMSE, with linear regression equation Y=12.182+0.046*X, and between SOD and MMSE, with linear regression equation Y=11.093+0.081*X. Furthermore, another cognition evaluation tools, MoCA was also applied in this study to confirm the relationship between Lp-PLA2/SOD, and results showed that a linear relationship was also found between Lp-PLA2/SOD and MoCA scores. The linear regression equation was Y=6.780+0.049*X between Lp-PLA2 and MoCA, and Y=2.103+0.111*X between SOD and MoCA scores (Fig. 4B, D).

### Lp-PLA2 and SOD as independent factors to predict CI in CSVD

Ordinal logistic regression and generalized linear models were used to examine the association between the two biomarkers and the extent of cognitive impairment. The results showed that relatively low levels of Lp-PLA2 (OR 0.977, *p*=0.003) and SOD (OR 0.933, *p*=0.001) were significant independent risk factors for cognitive impairment in CSVD patients as shown in Table 2. The predictive value of other variables, such as education and urea are also shown in Table 2.

### ROC analysis of the utility of Lp-PLA2 and SOD levels in the diagnosis of CSVD with CI

**Figure 5.**
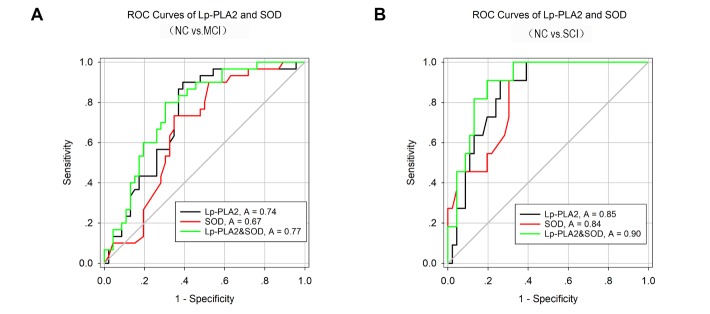
ROC analysis of Lp-PLA2 and SOD (**A**) Lp-PLA2 and SOD for CSVD patients with MCI versus CSVD patients with NC. The combined graph showed the Lp-PLA2 (black curve), SOD (red curve) and the combination curve (green curve) respectively. The combination of Lp-PLA2 and SOD was the best discriminating parameters compared to Lp-PLA2 and SOD alone. (**B**) ROC analysis of Lp-PLA2 and SOD for CSVD patients with SCI versus NC. The AUC was 0.85 for Lp-PLA2 (black curve), 0.84 for SOD (red curve) and 0.90 for the combination of Lp-PLA2 and SOD (green curve). Abbreviations: CSVD, Lp-PLA2, Lipoprotein-associated Phospholipase A2; SOD, Superoxide Dismutase; MCI, Mild Cognitive Impairment; SCI, severe cognitive impairment.

Due to the interaction between Lp-PLA2 and SOD, the ability of the combination of these two variables to serve as a better diagnostic value was examined. ROC curves were constructed, and the areas under the curves (AUCs) were calculated to assess the value and accuracy of Lp-PLA2 and SOD for discriminating CSVD patients with MCI from those CSVD patients with NC. The AUCs for Lp-PLA2 and SOD to discriminate between NC and MCI were 0.737 and 0.670, respectively; however, the combination of Lp-PLA2 and SOD increased the AUC to 0.770. The Youden index was 1.5087 for Lp-PLA2, 1.3783 for SOD, and 1.4957 for their combination (Fig. 5). The value of Lp-PLA2 and SOD for discriminating CSVD patients with SCI from those CSVD patients with NC was also evaluated. The AUCs for Lp-PLA2, SOD, and their combination to discriminate between NC and SCI were 0.855, 0.841. 0.899, respectively, while the Youden indices were 1.6483, 1.6739, and 1.7134. The sensitivity and specificity for Lp-PLA2 alone was 90.91% and 73.92%, respectively; however, combined with SOD, the specificity increased to 80.43% (Fig. 5).

## DISCUSSION

Inflammation plays a crucial role in the pathogenesis of cerebral ischemia including CVSD and dementia such as AD and MCI [2, 20, 21]. Plasma levels of vascular and neuroinflammation-related markers including Lp-PLA2 and SOD have been used as specific measures of cerebrovascular inflammation in ischemic stroke. Although the role of lipoprotein-associated phospholipase A2 in cognitive impairment is known; however, the role of SOD in cognitive impairment especially in CSVD patients remains largely unexplored. In this study, for the first time to our knowledge, we found the relation of Lp-PLA2 and SOD with cognitive impairment in CSVD patients. Our findings provide several novel observations. First, we note that plasma Lp-PLA2 and SOD levels in CSVD patients with MCI or SCI were lower than those in CSVD patients with NC and that the two markers were positively correlated to the degree of cognitive impairment in CSVD patients. Second, our study suggested that Lp-PLA2 and SOD may be closely correlated to the severity of white matter lesion in CSVD patients. Third, multiple linear regression showed a significant association between Lp-PLA2/SOD and the degree of cognitive impairment as measured by the MMSE and MoCA scores in CSVD patients, independent of LDL-C. Fourth, we further confirmed that Lp-PLA2 and SOD were significant independent risk factors for cognitive impairment in CSVD patients as examined by OLRGLMs. Finally, this study created Lp-PLA2 and SOD-related ROC curves to distinguish CSVD patients with MCI from those with NC. Together, these data support the integral roles of the vascular and neuroinflammatory markers Lp-PLA2 and SOD in CSVD patients with cognitive impairment.

Lp-PLA2 is a recognized marker of systemic vascular and neuro inflammation and cerebral/cardiovascular risk, which has been related to ischemic stroke and vascular dementia [22, 23]. The differences of blood Lp-PLA_2_ might be important, as plasma Lp-PLA_2_ levels change dynamically early after a cerebral-vascular event and reflect its action in the brain [24]. Several recent studies have examined Lp-PLA2 levels in the acute period after transient ischemic attack (TIA) or stroke and observed elevated levels of plasma Lp-PLA2, which were associated with early recurrent stroke [24, 25]. In this study, excluding other confounders (such as gender, age, years of education, BMI, hypertension, diabetes, CHD, medical statin use, total cholesterol, HDL-C, LDL-C, urea, Cr, UA and APOE), significantly lower levels of Lp-PLA2 in CVSD patients with MCI or SCI were observed compared to those in CVSD patients with NC (Fig. 2), and signifcant differences of Lp-LPA2 and SOD were noted among NC, MCI and SCI groups (Table 1). A significant correlation (Fig. 2C) between Lp-PLA2 and SOD was observed, suggesting that there might be a linkage between Lp-PLA2 and SOD in CSVD. These observations suggest that Lp-PLA2 might play important roles in preventing cognitive impairment development in CSVD. This result is consistent with previous findings that indicated antioxidative effects of Lp-PLA2 [26-28].

Recent findings have indicated that WMHs are associated with the cognitive impairment in CSVD [4, 29-31], and our results (Fig. 3D, E) are consistant with these findings. Furthermore, we note that the lower Lp-PLA2/SOD levels in the severe WMH CSVD patients (Fazekas 3 group) were found compared to those in the mild white matter lesion group (Fazekas 1 group; Fig. 3), stronlgy implying that lower levels of Lp-PLA2/SOD may mediate cognitive impairment in CSVD patients, probably partially via white matter lesion and lacunes. Based on this finding, we ask whether LP-PLA2 and SOD are correlated to cognitive impairment, and whether their lower levels are independently associated factors in mediating cognitive impairment in CSVD patients.

Some prior studies have suggested that the association between Lp-PLA2 and vascular events is strongest in patients with lower LDL-C levels [32] and have found a strong correlation between LDL-C and Lp-PLA2 in recurrent vascular events [24]. In contrast, another study found no significant difference in the predictive value of Lp-PLA2 based on baseline LDL-C level [33]. In our study, there was evidence of variability in the strong correlation between Lp-PLA2 and cognitive impairment in CSVD patients, which was independent of circulating LDL-C levels (Fig. 4A, B; Table 2), directly indicating that circulating LDL-C levels in CSVD patients with cognitive impairment may not influence or participate in the disease pathogenesis but rather that circulating Lp-PLA2 may influence CSVD patients via regulating vascular and neuroinflammatory mechanisms but not lipid metabolism.

The relationship between Lp-PLA2 and cognitive impairment reported in other studies has been inconsistent. Savas et al., indicated that Lp-PLA2 levels is higher in healthy subjects compared to AD patients, implying that Lp-PLA2 may play an anti-inflammatory or anti-oxidative effects in AD [34]. A small cross-sectional study of 78 AD cases, 59 amnestic MCI cases, and 66 cognitively normal controls performed by Davidson et al., did not find a significant association between Lp-PLA2 and AD [35]. One prospective Rotterdam study and Texas Alzheimer’s Research and Care Consortium case-control study showed that the levels of Lp-PLA2 were associated with an increased risk of vascular dementia (VD) and AD, independent of other cardiovascular disease (CVD) and inflammatory factors [23, 36]. The discrepancy between our findings and other studies may be explained by differences in study design, different selected subjectes, data collection, and the number of variables used in the adjusted models to determine in these studies. In the current study, CSVD but not AD patients were chosen, and we only measured Lp-PLA2 mass. Lp-PLA2 mass detects a portion of the total Lp-PLA2, mainly the Lp-PLA2 associated with HDL, which is the “good cholesterol” that displays protective effects [16]. In the current study, in the plasma we did not noted any differences of systemically infection-related inflammatory mediators (such as a leukocyte, hs-CRP, neutrophile granulocyte, ESR and uric acid; Table 1) between CSVD patients and healthy subjects. Adjust for those infective and inflammatory potential confounders, and due to the nature and changes in circulating Lp-PLA2 in CSVD patients, our findings suggest that peripheral vascular or neuroinflammatory damage may be a key mediating mechanism by which Lp-PLA2 predisposes CSVD patients to cognitive impairment.

In addition, we observed a similar trend upon examination of the levels of plasma SOD across CSVD groups (Fig. 4C, D), strongly suggesting that lower levels of SOD may be correlated to cognitive impairment, and the lower SOD level is independent risk factors in mediating cognitive impairment in CSVD patients. SOD levels in CSVD patients move in a similar direction as the levels of Lp-PLA2, further confirming that some anti-inflammatory and proinflammatory-related markers may behave similarly in certain circumstances. SOD is recognized as an antioxidative stress and anti-neuroinflammatory-related mediator [37-39]. The gradual downregulation of SOD levels in CSVD patients according to the severity of cognitive impairment and the significant positive correlation between SOD levels and the degree of cognitive impairment strongly suggest that more circulating oxidative stress and inflammatory markers may be present in CSVD subjects with cognitive impairment than in those with NC. These data suggest that plasma SOD levels are not increased in these patients with cognitive impairment; rather, the decline in SOD levels indicates a deficit in antioxidant defense mechanisms in those CSVD subjects with cognitive impairment, since these CSVD patients are unable to remove the circulating superoxide anion, therefore suffering an increase in small vascular damage induced by ROS and neuroinflammation. Thus, a lower level of plasma SOD is associated with increased vascular damage. Due to the retrospective nature of this study, we cannot exclude the possibility that CSVD patients with cognitive impairment produce less SOD and/or that CSVD patients with NC produce more SOD. However, longitudinal studies may provide a clue as to whether Lp-PLA2 and SOD play crucial roles in the development of cognitive impairment in subjects with CSVD.

Our results further indicate that only Lp-PLA2 and SOD but not hypertension, UA, Cr, HDL-C, or LDL-C are independent predictors of cognitive impairment in patients with CSVD (Table 2). This interesting finding demonstrates that Lp-PLA2 and SOD may directly participate in the pathogenesis of cognitive impairment in CSVD via regulating vascular and neuroinflammatory damage in circulating blood. Since both Lp-PLA2 and SOD are involved in the neuropathogenesis of CSVD patients and only some but not all CSVD subjects display cognitive impairment, we attempted to determine whether Lp-PLA2 and SOD have enough discriminative power to distinguish CSVD patients with cognitive impairment from those with NC. ROC curve analysis revealed an AUC of 0.737 for Lp-PLA2 and 0.670 for SOD, indicating that both had an acceptable sensitivity and specificity for the potential discrimination of CSVD patients with MCI from those with NC, and Lp-PLA2 had better discrimination accuracy than SOD. The ROC analysis strongly suggests that Lp-PLA2 and SOD can significantly discriminate CSVD patients with MCI from those with NC and therefore could be used as a valuable diagnostic tool in early screening for cognitive impairment in CSVD subjects. Moreover, the combination of Lp-PLA2 and SOD produced a larger AUC of 0.770 with a sensitivity of 80% and specificity of 69.7% in the ROC analysis than Lp-PLA2 or SOD alone, strongly implying that the diagnostic accuracy of the combination of the two variables was superior to that of either variable alone in differentiating CSVD patients with different cognitive status.

The present study is limited mainly by its retrospective nature, assessing plasma levels of Lp-PLA2 and SOD in a relatively small sample size of CSVD patients at a single time point. Prospective and longitudinal studies are needed, and randomized trials of potent reversible pharmacological Lp-PLA2 inhibitors or SOD agonists may help to clarify whether modification of Lp-PLA2 or SOD can delay the progression of CSVD.

A study strength is that there is very limited evidence that Lp-PLA2 and SOD, especially SOD, are significant and independent predictors associated with cognitive impairment in CSVD patients. The observed association of Lp-PLA2 and SOD with CSVD in our study is consistent with the hypothesis that Lp-PLA2 and SOD, as measured in peripheral blood, may reflect vascular and neuroinflammatory mechanisms. The relationship between Lp-PLA2/SOD and CSVD is highly likely to be mediated at least partially via vascular and neuroinflammatory mechanisms. These findings may be important for health care facilities that have limited access to Neurologists specialized in the diagnosis of CSVD with cognitive impairment. Further research in these areas is of great importance because Lp-PLA2 and SOD are modifiable risk factors and, if further confirmed, may be considered as therapeutic targets for preventing cognitive impairment development in CSVD and reducing its severity.

## Supplemetary Materials

The Supplemenantry data can be found online at: www.aginganddisease.org/EN/10.14336/AD.2019.0304


