# Supplementary material for: Plasma Lipoprotein-associated Phospholipase A2 and Superoxide Dismutase are Independent Predicators of Cognitive Impairment in Cerebral Small Vessel Disease Patients: Diagnosis and Assessment
Source: Aging Dis. 2019 Aug 1;10(4):834–46. doi: 10.14336/AD.2019.0304 (PMC6675532; doi:10.14336/AD.2019.0304)
Supplement: Supplementary file 1 [file AD-10-4-834-s.pdf]

SUPPLEMENTARY DATA

Plasma Lipoprotein-associated Phospholipase A2 and Superoxide Dismutase are Independent Predicators of Cognitive Impairment in Cerebral Small Vessel Disease Patients: Diagnosis and Assessment

Shuzhen Zhu[1,#], Xiaobo Wei[1,#], Xiaohua Yang[1,#], Zifeng Huang[1,#], Zihan Chang[1], Fen Xie[1], Qin Yang[1], Changhai Ding[2, 3], Wei Xiang[4], Hongjun Yang[4], Ying Xia[5], Zhong-Ping Feng[6], Hong-Shuo Sun[6,7], Midori A. Yenari[8], Lin Shi[9, 10], Vincent CT Mok[11,*], Qing Wang[1,*]

[1]Department of Neurology and [2]Department of Orthopedics, Zhujiang Hospital of Southern Medical University, Guangdong, China. [3]Menzies Institute for Medical Research, University of Tasmania, Hobart, Australia. [4]Department of Neurology, Guangzhou General Hospital of Guangzhou Military Command, Guangdong, China. [5]Shanghai Key Laboratory of Acupuncture Mechanism and Acupoint Function, Fudan University, Shanghai, China. [6]Department of Physiology and [7]Department of Surgery, Faculty of Medicine, University of Toronto, Toronto, Ontario M5S 1A8, Canada. [8]Department of Neurology, University of California, San Francisco & the San Francisco Veterans Affairs Medical Center, San Francisco, USA. [9]Department of Imaging and Interventional Radiology, The Chinese University of Hong Kong, Hong Kong, China. [10]BrainNow Research Institute, Shenzhen, China. [11]Gerald Choa Neuroscience Centre, Department of Medicine and Therapeutics, Faculty of Medicine, Prince of Wales Hospital, The Chinese University of Hong Kong, Hong Kong, China.

© 2019. Zhu S et al. Published online at http://www.aginganddisease.org/EN/10.14336/AD.2019.0304

SUPPLEMENTARY DATA

Supplementary Table 1. Linear correlation of Lp-PLA2/SOD with MMSE scores.

**Variable**	**Multivariable ***	
	**β**	**p value**
Lp-PLA2	0.291	0.002**
SOD	0.344	0.000***
Education	0.231	0.012*
Age	-0.147	0.115
BMI	0.036	0.688
Gender	-0.138	0.121
Cholesterol	0.061	0.516
HDL-C	-0.065	0.470
LDL-C	-0.011	0.904
Urea	-0.100	0.904
Cr	-0.122	0.177
UA	-0.002	0.982
Hypertension	0.062	0.481
Diabetes	0.009	0.917
CHD	-0.005	0.957
Statin	0.104	0.231
Antithrombotic	0.160	0.064
APOE4	-0.024	0.786

Multiple linear regression models with backwards elimination showed a positive linear correlation between Lp-PLA2/SOD and MMSE scores after adjusting for confounders as showed in the table. Dependent variables: MMSE Scores; independent variable: Lp-PLA2 and SOD. Covariates with **p*-value <0.05, ** *p* <0.01, *** *p* <0.001 are presented in bold. Abbreviations: BMI, body mass index; CHD, coronary heart disease; HDL-C, High-density lipoprotein cholesterol; LDL-C, low-density lipoprotein cholesterol; Cr, creatinine; UA, uric acid; Che, Cholinesterase; APOE, apolipoprotein E.

© 2019. Zhu S et al. Published online at http://www.aginganddisease.org/EN/10.14336/AD.2019.0304
